# Polymorphism of Sr_2_ZnIrO_6_ Double Perovskite at High Pressure and Temperature

**DOI:** 10.1021/acs.inorgchem.5c02651

**Published:** 2025-08-19

**Authors:** Virginia Monteseguro, Paula Kayser, Marina Teresa Candela, Samuel Gallego-Parra, Catalin Popescu, Juan Ángel Sans, José Antonio Alonso, Javier Ruiz-Fuertes

**Affiliations:** † DCITIMAC, 16761 Universidad de Cantabria, Avenida Los Castros 48, E-39005 Santander, Spain; ‡ Departamento de Química Inorgánica, Facultad Químicas, Universidad Complutense de Madrid, Avda. Complutense s/n, Ciudad Universitaria, 28040 Madrid, Spain; § Departamento de Física Aplicada, Universidad de Cantabria, Avda. Los Castros 48, 39005 Santander, Spain; ∥ Grupo de Nanomedicina, IDIVAL-Universidad de Cantabria, Avda. Cardenal Herrera Oria, 39011 Santander, Spain; ⊥ 55553 European Synchrotron Radiation Facility (ESRF), 71 Avenue des Martyrs, F-38000 Grenoble, France; # ALBA-CELLS Synchrotron Light Facility, E-08290 Cerdanyola del Vallès, Barcelona, Spain; ¶ Instituto de Diseño para La Fabricación y Producción Automatizada, MALTA Consolider Team, Universitat Politécnica de Valencia, 46022 Valencia, Spain; ∇ Instituto de Ciencia de Materiales de Madrid (ICMM), 69570 Consejo Superior de Investigaciones Científicas (CSIC), Sor Juana Inés de la Cruz 3, 28049 Madrid, Spain

## Abstract

The structural behavior of Sr_2_ZnIrO_6_ double perovskite has been systematically studied under extreme conditions, both at high temperature and high pressure with synchrotron powder X-ray diffraction. At high temperatures, three structural phase transitions are detected at 473, 673, and 873 K, giving the sequence *P*2_1_/*n* → *I*2/*m* → *I*4/*m* → *Fm*3̅*m*. At high pressure, a phase transition from monoclinic *P*2_1_/*n* to tetragonal *I*4/*m* symmetry is observed at 7.5(1) GPa, as confirmed by ab initio calculations. The tetragonal high-pressure *I*4/*m* polymorph appears at both high and intermediate temperatures, highlighting a displacive transition with an estimated negative pressure dependence of the critical temperature (d*T*_c_/d*p* = −51 K/GPa). The bulk modulus (∼150 GPa) and the volumetric thermal expansion (∼3.4 × 10^–5^ K^–1^) are determined for the low- and high-pressure phases (*P*2_1_/*n* and *I*4/*m*).

## Graphical abstract

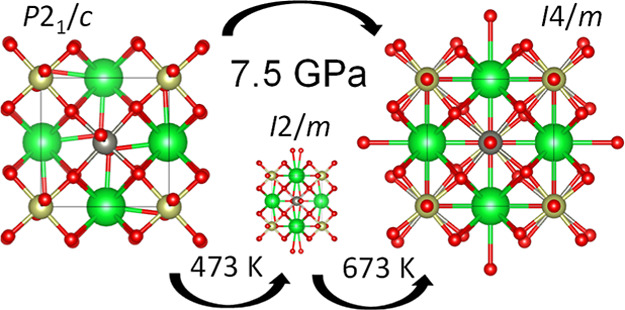

## 1 Introduction

Iridium oxides exhibit fascinating electronic and magnetic properties due to the interplay between comparable energy scales of spin–orbit coupling (SOC), crystal field (CF) effects, electronic bandwidth, and Coulomb and exchange interactions. According to theoretical predictions, some of them behave as different types of Mott insulators,^1^ others are Weyl semimetals,^2^ and a few of them are Hall spin systems.^3^ Regarding the technological applications of these compounds, some iridium oxides have been recently proposed^4^ for proton exchange membrane water electrolysis, thereby facilitating the sustainable production of green hydrogen.

The family of Sr_2_ *M*IrO_6_ compounds belongs to the *A*_2_ *BB*′O_6_ double-perovskite-type structure with 1:1 rock-salt ordering over the *B*-site described in monoclinic space group *P*2_1_/*n* (Figure 1a). These oxides show exceptional electronic versatility, allowing the valence of iridium to switch between Ir^4+^, Ir^5+^, and Ir^6+^, depending on the oxidation state of the *M* cation. This, in turn, can result in variations in the Ir–O bond’s covalency across different family members. To date, the majority of Sr_2_ *M*IrO_6_ compounds containing Ir^6+^ (Mg, Ca, Zn, and Ni) crystallize in a *P*2_1_/*n* monoclinic structure (*Z* = 2), in which Ir and *M* occupy distorted octahedral sites forming two fcc interpenetrating lattices of [Ir^6+^O_6_] and [*M* ^2+^O_6_] (Figure 1a).

**Figure 1:**
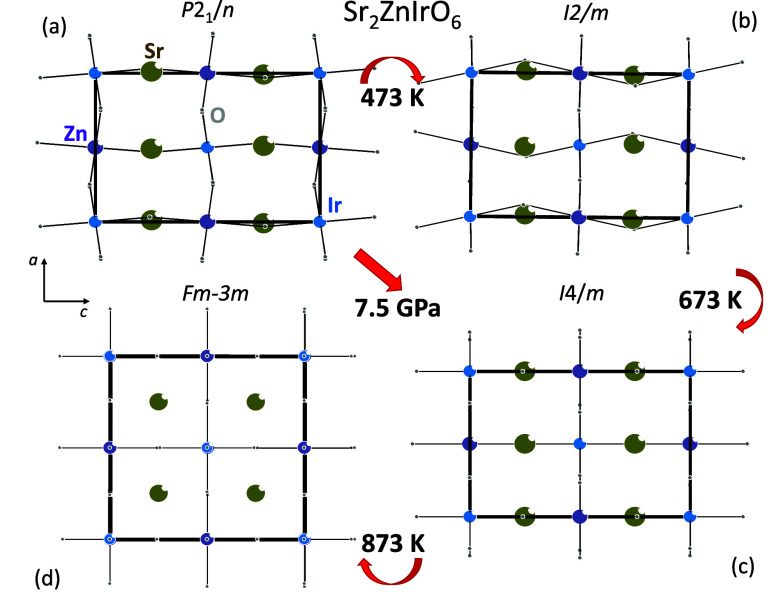
Crystal structures of the different polymorphs of Sr_2_ZnIrO_6_. In order to facilitate the visualization, only the Ir–O and Zn–O bonds are shown. Sr, Zn, Ir, and O are represented by brown, purple, blue, and gray balls, respectively. The monoclinic *P*2_1_/*n* structure at ambient conditions (a) transforms at ∼473 K to monoclinic *I*2/*m* (b), at ∼673 K transforms to tetragonal *I*4/*m* (c), and finally at ∼873 K transforms to cubic *Fm*3̅*m* (d). At 7.5 GPa, the transition occurs directly from monoclinic *P*2_1_/*n* (a) to tetragonal *I*4/*m* (c).

Considering the low symmetry of their structures, any variation in the Ir–O, Ir–Ir, or Ir–*M* environment through compression or expansion is likely to induce structural transitions that significantly change the SOC and CF and therefore the electronic structure and magnetism of these compounds.

In a recent high-pressure X-ray absorption spectroscopy (XAS) and powder X-ray diffraction (XRD) study,^5^ it was observed that the absorbed intensity of the circularly polarized spectrum in the Ir L_3_-edge of the Sr_2_NiIrO_6_ perovskite shows an abrupt intensity change around 18 GPa, concomitant with a discontinuity of the *b*/*a* lattice parameters ratio. Such a change has been explained in terms of the variation in the strength of the long-range antiferromagnetic Ir–Ir interaction resulting from a *P*2_1_/*n* to *I*1̅ structural phase transition. At a high temperature, a previous neutron diffraction study^6^ shows that Sr_2_NiIrO_6_ undergoes the following phase transition sequence: first, from monoclinic *P*2_1_/*n* to tetragonal *I*4/*m* at 473 K and second from tetragonal *I*4/*m* to cubic *Fm*3̅*m* at 673 K.

Studies of the Ir–Ir antiferromagnetic coupling in double perovskites have revealed that, in both Sr_2_NiIrO_6_ and Sr_2_ZnIrO_6_ compounds,^7^ the Ir–Ir exchange interaction is unexpectedly stronger than the Ir–Ni or the Ir–Zn one. Therefore, investigating this behavior in the different polymorphs of this family compounds would be essential to fully understand such a strong Ir–Ir magnetic interaction. Although the polymorphism of Sr_2_NiIrO_6_ is well studied under high pressure and temperature, the structural behavior of Sr_2_ZnIrO_6_ is practically unexplored under extreme conditions. There exists just one high-pressure work reporting the emergence of an unexplained feature above 7 GPa in the XAS spectrum of the L_3_-edge of Ir in Sr_2_ZnIrO_6_, which might be consistent with an abrupt increase of the IrO_6_ CF.^5^

In this work, we performed high-temperature XRD and high-pressure XRD to study the polymorphs of Sr_2_ZnIrO_6_ up to 1273 K and 16 GPa. In addition, we carried out ab initio calculations to study the stability of the low- and high-pressure structures of Sr_2_ZnIrO_6_.

## 2 Experimental and Calculation Details

A polycrystalline Sr_2_ZnIrO_6_ sample was synthesized from highly reactive precursors obtained through the Pechini method.^6,7^ Stoichiometric amounts of 0.004 mol of Sr­(NO_3_)_2_ (Merck) and 0.002 mol of ZnO (Alfa Aesar 99.999%) were dissolved in 100 mL of citric acid aqueous solution (10% w/w) with 1 mL of HNO_3_. IrO_2_ (Strem 99%) (0.002 mol) was added to this solution and remained in suspension with continuous stirring; nevertheless, this did not hinder the formation of highly homogeneous samples, with iridium fully incorporated into the double perovskite structure. The suspension was slowly evaporated, and the resulting organic resin was dried at 140 °C. Following this, the material was initially heated in air at 600 °C for 12 h at a rate of 2 °C/min to decompose organic components and eliminate residual nitrates. To stabilize the Ir^6+^ oxidation state, further thermal treatments under oxidizing conditions were performed. First, the resulting precursors were ground and calcined in an O_2_ flow at 900 °C for 12 h. Then, after a second grinding, the product was subjected to a high-pressure thermal treatment under 200 bar of oxygen at 900 °C for 48 h. The progress of the reaction was monitored by X-ray diffraction (XRD), using a Bruker-AXS D8 diffractometer (40 kV, 30 mA), controlled by the DIFFRACPLUS software, with Bragg–Brentano geometry (Cu Kα, λ = 1.5418 Å) and a position-sensitive detector. The result was a highly pure Sr_2_ZnIrO_6_ sample in the *P*2_1_/*n* space group with 1.86(6)% of ZnO present as impurity.

High-angular resolution experiments of synchrotron powder X-ray diffraction (S-XRD) data were performed at the MSPD beamline of the ALBA-CELLS Synchrotron. The S-XRD patterns were collected in the high-resolution MAD setup^8^ using a wavelength of 0.3251 Å (38 keV) over the angular range 1° < 2θ < 38°. This wavelength gives access to a large *Q* range and gives an optimal sample absorption. The samples were sealed in 0.7 mm-diameter quartz capillaries, which were rotated during the measurements and transmission geometry was used. Temperature control was achieved using an FMB Oxford hot air blower (RT-1223 K) on the sample in situ. Diffraction data were collected for 1 h at 473, 673, 873, 1073, and 1273 K.

Structural refinements, using the Rietveld method,^9^ were carried out using the FULLPROF refinement program.^10^ A pseudo-Voigt function was chosen to generate the line shape of the diffraction peaks. No regions were excluded from the refinement. The following parameters were refined in the final analysis: scale factor, zero-point error, background (24-term shifted Chebyshev) coefficients, lattice parameters, positional coordinates, and isotropic atomic displacement.

Two high-pressure XRD experiments were performed. In run 1, the experiment was carried out at the ALBA-CELLS synchrotron (MSPD beamline)^8^ (λ = 0.4246 Å, beam size 20 μm^2^ × 20 μm^2^), and in run 2, the experiment was done at the European Synchrotron Radiation Facility (ESRF) synchrotron (ID15B beamline),^11^ (λ = 0.41 Å, beam size 5 × 5 μm^2^). At ALBA-CELLS, a Rayonix SX165 CCD was used, while at the ESRF, we employed an EIGER2 × 9 M CdTe flat panel detector. In run 1, a Boehler-ALMAX diamond anvil cell (DAC) equipped with 350 μm culet diamonds was used to generate pressure. In between both diamonds, a stainless-steel gasket preindented to 40 μm and drilled with a hole of 150 μm was placed. Inside the gasket hole, 20 μm-thick pellets of compressed sample powder were loaded with a ruby chip (pressure calibrant),^12^ and Ne was the pressure-transmitting medium (PTM) in the pressure chamber. In this experiment, the maximum pressure reached was 16.9 GPa. In run 2, a membrane-type DAC was employed with 600 μm culet diamonds mounted. Between both diamonds, we placed a stainless-steel gasket preindented to 90 μm, with a 300 μm hole diameter as the pressure chamber. Ruby chips were used as pressure calibrants, and in this case, a mixture of 4:1 methanol–ethanol was employed as PTM. This experiment was simply used to obtain more pressure points in the 1 atm to 5 GPa being terminated at this pressure.

The stability of the high-pressure polymorph of Sr_2_ZnIrO_6_ found by XRD with respect to the low-pressure structure was tested with the Vienna ab initio simulation package within the density functional theory formalism.^13–16^ The calculations were carried out at *T* = 0 K using the pseudopotential method and the projector augmented wave scheme. Highly converged results were achieved by extending the set of plane waves up to a kinetic energy cutoff of 500 eV. The local spin-density approximation, GGA + U, in the Dudarev’s approach^16^ was used to correctly describe the strongly correlated 3d and 5d electrons of zinc and iridium, respectively. The effective Hubbard potential *U*_eff_ was set to 1 eV for Zn and 3 eV for Ir ions, yielding reliable results of the lattice parameters and magnetic moments compared to the experimental ones. This structure was relaxed taking into account its antiferromagnetic character at low temperatures.^17^ We have used the PBE description^18^ within the GGA approximation for the exchange–correlation energy. At each selected volume, the structure was fully relaxed to its equilibrium configuration through calculation of the forces on atoms and of the stress tensor. A dense 8 × 8 × 6 *k*-point mesh was used to perform integrations within the Brillouin zone. In the relaxed configurations, the forces on the atoms are less than 0.006 eV Å^–1^, and the deviation of the stress tensor from a diagonal hydrostatic form is less than 0.1 GPa. The agreement between the experimental and calculated lattice parameters of Sr_2_ZnIrO_6_ at ambient pressure (Table 1) with deviations below 1% demonstrates the reliability of our calculations.

**Table 1:** Comparison of the Experimental and Calculated Lattice Parameters and Ir–O–Zn Tilting Angle along the [001] Direction at Ambient Pressure for Sr_2_ZnIrO_6_

	exp.	calc.
*a* (Å)	5.6216(1)	5.5803
*b* (Å)	5.5856(1)	5.5604
*c* (Å)	7.9052(2)	7.8631
β (°)	90.01(1)	90.082
Φ[001] (°)	9.8(2)	9.12

## 3 Results and Discussion

### 3.1 High Temperature

In Figure 2, we show the high-temperature XRD patterns that were acquired at 473, 673, 873, 1073, and 1223 K (with the room temperature pattern included for comparison) to study the thermal evolution of the crystal structure. An initial visual analysis of the data indicates the presence of structural phase transitions to higher symmetries, such as tetragonal or cubic, as the peak splitting seems to vanish with increasing temperature.

**Figure 2:**
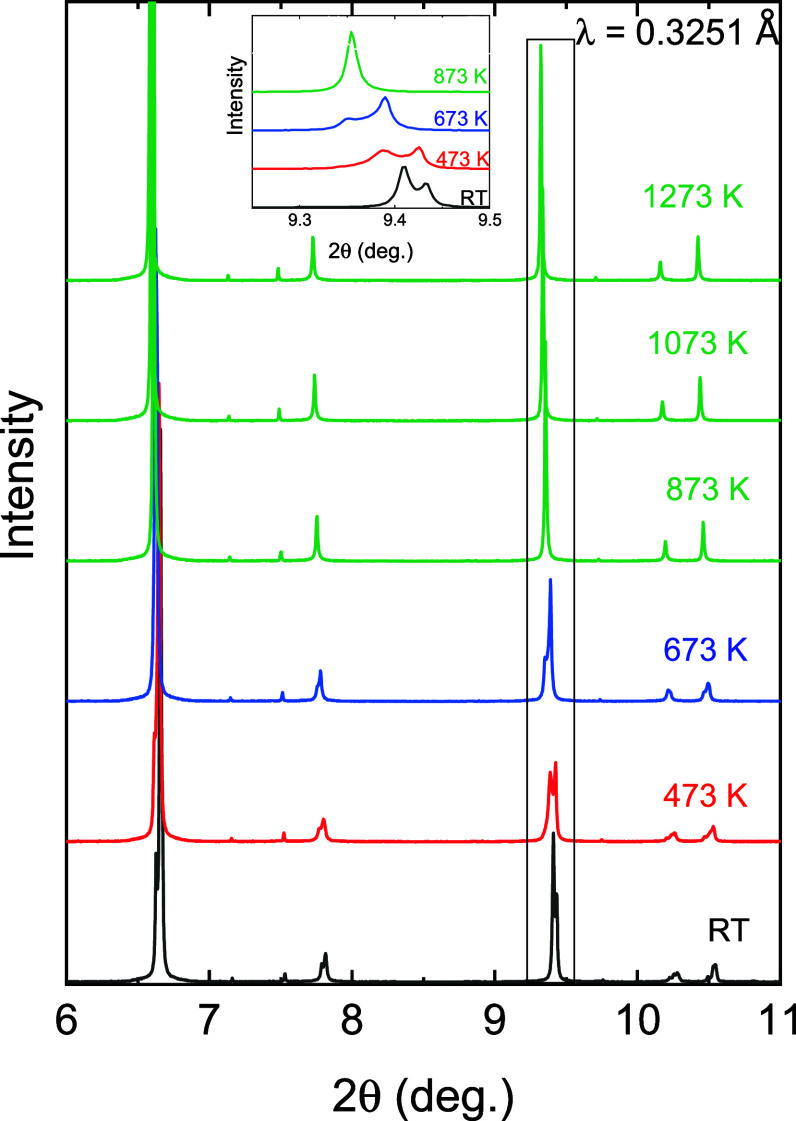
Collection of powder diffraction patterns of Sr_2_ZnIrO_6_ obtained at different temperatures. The changes observed in the position and number of reflections indicate up to three structural phase transitions. The inset shows a zoom of the 9.25° to the 9.5° 2θ-range to clarify the occurrence of the phase transition and the expansion of the lattice with temperature.

The Rietveld refinements are shown in Figure 3. As explained in the Introduction and shown in Figure 1, at RT, the structure crystallized in the monoclinic space group *P*2_1_ */n*; this system, represented by the Glazer notation (*a* ^*–*^ *a* ^*–*^ *c* ^*+*^), exhibits out-of-phase tilting along the [100] and [010] directions while demonstrating in-phase tilting along the [001] direction with respect to the pseudocubic perovskite unit cell. Upon heating, three phase transitions have been identified following this sequence: *P*2_1_/*n* → *I*2*/m* → *I*4/*m* → *Fm*3̅*m* similarly to the sequence observed in Sr_2_NiIrO_6_ as a function of temperature.^6^ This completes the evolution described before^6^ from monoclinic to tetragonal and finally to cubic in the 373–673 K range, from a neutron powder diffraction study. The former transformation has been detected at 473 K, showing a structural evolution to monoclinic space group *I*2*/m*. The second-order phase transition from *P*2_1_/*n* to *I*2*/m* provokes the annulation of out-of-phase octahedral tilting along the direction [001] of Figure 1b. A further increase in temperature leads to a second phase transition, which has been identified based on the *c*/*a* pseudotetragonal ratio. It undergoes a reversal from *c*/*a* < 1 at 473 K to *c*/*a* > 1 at 673 K. It indicates a reorientation of the octahedral tilting, changing form the (*a* ^*–*^ *a* ^*–*^ *c* ^0^) system in the monoclinic *I*2/*m* symmetry to the (*a* ^0^ *a* ^0^ *c* ^*–*^) in the tetragonal *I*4/*m* space group (Figure 1c). Group theory indicates that this transition must be of the first order.^19^ The structural analysis at temperatures higher than 873 K reveals the presence of the cubic phase characterized with the (*a* ^0^ *a* ^0^ *a* ^0^) tilting system (Figure 1d). No additional changes are observed above 873 K apart from a position shift of the reflections toward lower 2θ angles as a result of the lattice expansion with temperature.

**Figure 3:**
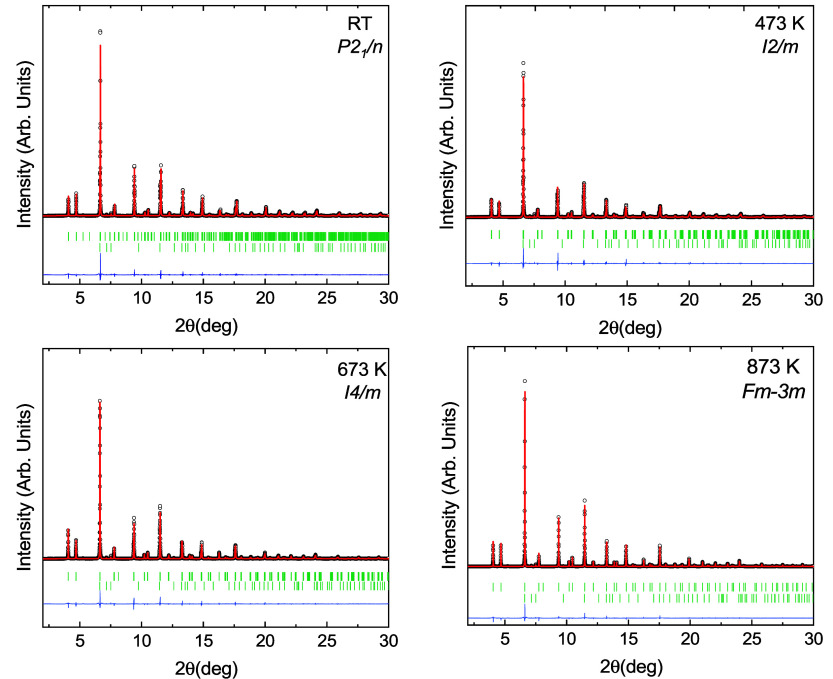
Diffraction patterns obtained for each structure with their Rietveld fit and the residual of the fits (continuous red and blue lines). The vertical green lines show the reflections positions for (top to bottom) Sr_2_ZnIrO_6_ and ZnO. The wavelength used in the experiments was 0.3251 Å.

Figure 1 displays a schematic view of the structures. Considering that the limited number of data points does not allow us to unequivocally identify the onset of the phase transitions, we shall consider that the phase transitions occur at the temperatures at which the new phases are identified in our experiment. It is observed that in the *P*2_1_/*n* to *I*2/*m* phase transition at 473 K, four of the Ir–O and Zn–O bonds of each polyhedron enter the (110) plane. Therefore, the other two Ir–O and Zn–O bonds forming Ir–O–Zn zigzag chains along the *c*-axis compress and the $\hat{IrOZn}$ angles change from 159° to 153°. The *I*2/*m* to *I*4/*m* phase transition at 673 K consists of the change of the $\hat{IrOZn}$ angles 153° to 180° and consequently aligns the Ir–O–Zn chains along the *c*-axis, producing an expansion of *c* and a drastic symmetry increase. Above 873 K, the polyhedra lose their tilts, and the structure becomes cubic in space group *Fm*3̅*m*.

The lattice parameters, atomic coordinates, thermal parameters, and fitting factors are summarized in Table 2, and the thermal evolution of the lattice parameters and unit-cell volume are plotted with temperature in Figure 4. There, it can be seen that the first transition at 473 K involves a relative contraction of the *c* axis followed by a large expansion of the *c* axis in the second transition at 673 K. The abrupt contraction and expansion of the *c*-axis in double perovskites is normally related to an increase followed by a decrease of the polyhedral tilting along this direction.^19^ Considering the possible monoclinic and tetragonal polymorphs found in other double perovskites, we rapidly identify that at 473 and 673 K, the diffraction pattern can be refined in the monoclinic *I*2/*m* (Figure 1b) and tetragonal *I*4/*m* (Figure 1c) space groups. Above 873 K, the structure is cubic and described in the space group *Fm*3̅*m* (Figure 1d).

**Table 2:** Space Group, Lattice Parameters, Atomic Coordinates, and Refinement Parameters of the Different Structural Transitions Found for Sr_2_ZnIrO_6_ at High Temperatures^a^

		*P*2_1_/*n*	*I*2/*m*	*I*4/*m*	*Fm*3̅*m*		
S.G		RT	473 K	673 K	873 K	1073 K	1223 K
	*a* (Å)	5.58750(4)	5.6023(1)	5.61809(5)	7.97382(3)	7.98981(4)	8.00156(4)
	*b* (Å)	5.62349(4)	5.63324(9)	5.61809(5)			
	*c* (Å)	7.90828(6)	7.9155(1)	7.97370(9)			
	β (°)	90.0029(4)	89.9940(8)				
	*V* (Å^3^)	248.488(3)	249.807(7)	251.674(4)	506.990(4)	510.047(4)	512.299(4)
	*Z*	2	2	2	4	4	4
Sr	*x*	0.01013(9)	0.5122(14)				
	*y*	0.49997(6)	0				
	*z*	0.25018(2)	0.2482(5)				
	*B* (Å^2^)	0.63(2)	0.77(4)	1.25(2)	1.62(2)	1.97(2)	2.22(2)
Zn	*B* (Å^2^)	0.17(3)	0.34(6)	0.73(3)	1.01(3)	1.16(3)	1.30(4)
Ir	*B* (Å^2^)	0.40(1)	0.46(2)	0.53(1)	0.83(1)	1.04(1)	1.21(2)
O1	*x*	0.0316(5)	0.9168(4)	0			
	*y*	0.05633(19)	0	0			
	*z*	0.24320(20)	0.2445(2)	0.2408(1)			
	*B* (Å^2^)	1.18(16)	1.9(2)	2.5(2)	3.1(1)	3.5(1)	3.8(1)
O2	*x*	0.2521(5)	0.2425(2)	0.268(3)			
	*y*	0.2285(4)	0.2469(2)	0.219(3)			
	*z*	0.0240(10)	0.0042(4)	0			
	*B* (Å^2^)	1.18(16)	1.9(2)	2.45(16)			
O3	*x*	0.2350(5)					
	*y*	0.7452(4)					
	*z*	0.9755(10)					
	*B* (Å^2^)	1.18(16)					
Reliability Factors							
χ2		6.93	8.59	7.03	9.73	11.0	11.8
Bragg *R*-factor		4.46	5.20	4.08	4.55	4.97	5.44
Rf-factor		4.94	3.69	3.28	4.33	4.90	5.36

a The formula units per unit cell are also shown to facilitate the comparison of the unit-cell volumes.

**Figure 4:**
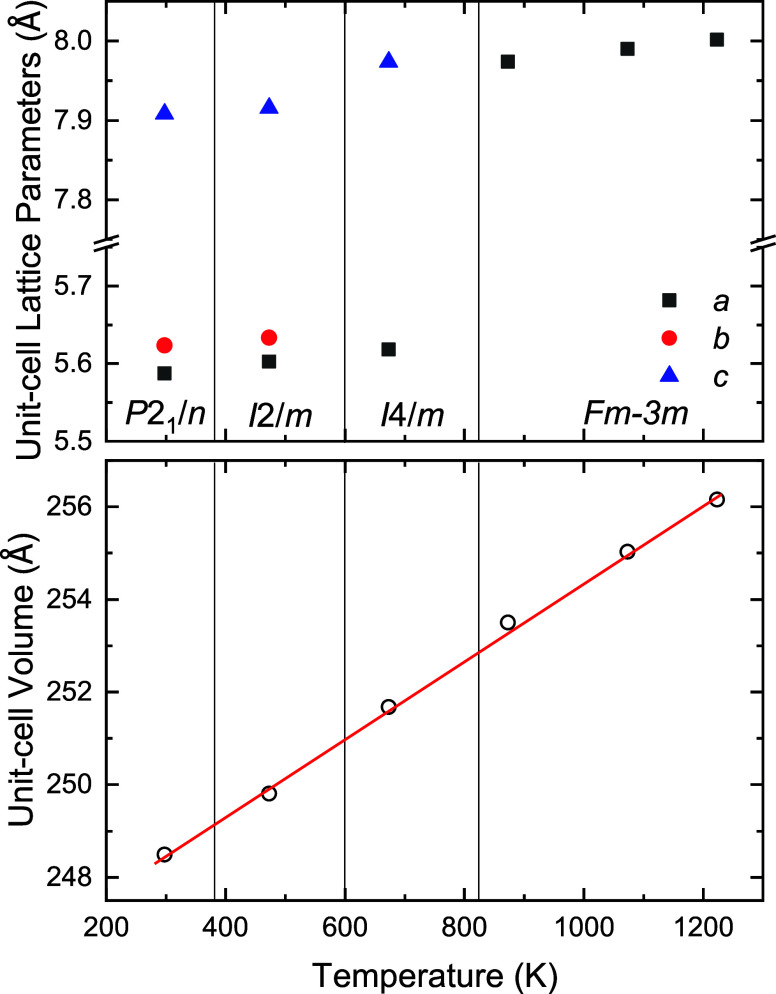
Temperature dependence of the lattice parameters and unit-cell volume of Sr_2_ZnIrO_6_, as determined from Rietveld refinements of the diffraction patterns. The vertical lines indicate tentative estimates of the stability ranges of the different polymorphs, assuming no phase coexistence. Open circles represent the unit-cell volume normalized to *Z* = 2 for all structures to facilitate comparison. The continuous red line corresponds to the linear fit applied to the unit-cell volumes, which gives rise to an average volumetric thermal expansion of ∼3.4 × 10^–5^ K^–1^.

### 3.2 High Pressure

A collection of powder XRD diffractograms of Sr_2_ZnIrO_6_ is presented in Figure 5. At low pressures, all reflections can be indexed to the initial *P*2_1_/*n* structure except for minor peaks that may be mistaken for noise. These arise from the 1.86(6)% ZnO impurity present in the sample and from solid neon reflections, which appear upon solidification above 6 GPa.

**Figure 5:**
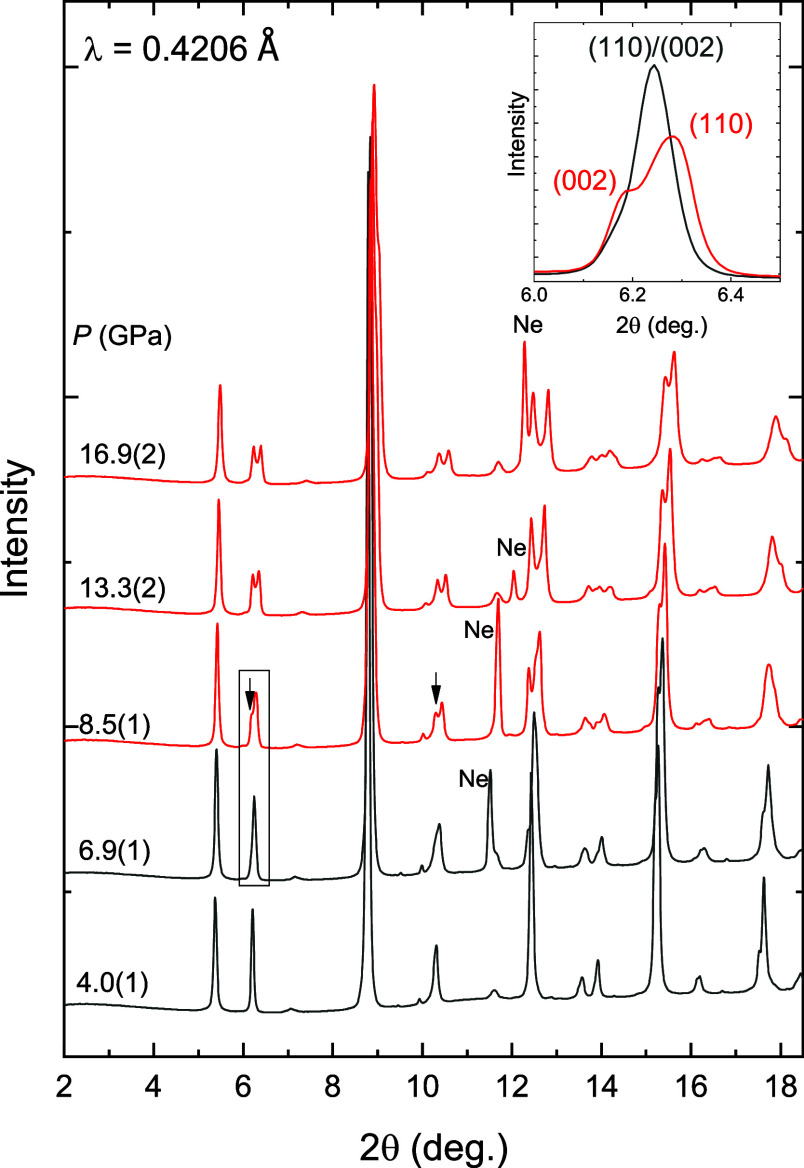
Diffraction patterns of Sr_2_ZnIrO_6_ at different pressures. The reflections that appear when the pressure transmitting medium (Ne) solidifies are identified. Arrows indicate the emergence of additional shoulders as a result of the structural phase transition. The reflections around 6° at 6.9(1) and 8.5(1) GPa are shown at the inset to clarify the occurrence of the phase transition.

With pressure, up to 6.9(1) GPa, reflections move toward higher two-theta angles as a result of a shortening of bond distances. Up to that pressure, all the reflections can be indexed with the starting monoclinic structure in space group *P*2_1_/*n*. From 7.5(1) GPa, some reflections start to split, and the whole diffraction pattern can be indexed with a tetragonal lattice. Such changes indicate a structural phase transition which coincides in pressure (7 GPa) with the change found in the XAS signal in Sr_2_ZnIrO_6_ in a previous work^5^ possibly due to a change in the local structure of the IrO_6_ polyhedra.

To identify the high-pressure phase of Sr_2_ZnIrO_6_, it is necessary to follow the evolution of the octahedral tilting angle (ϕ) under compression in order to extrapolate the structural behavior, where ϕ = (180 – $\hat{ZnOIr}$ )/2.^6^ However, the limited access to a maximum of 2θ = 18.2° due to the DAC opening, combined with the used wavelength and the overlapping reflections from several families of planes in the *P*2_1_/*n* symmetry due to the coincidence in *d*-spacing, prevents any reliable Rietveld refinement. For this reason, the procedure to find the high-pressure phases of this compound consisted of testing all the monoclinic and tetragonal polymorphs previously reported in the *A*_2_ *BB*’O_6_ double perovskites. The best intensity coincidence was found for the Sr_2_FeMoO_6_-type *I*4/*m* structure^20^ depicted in Figure 1c. This is exactly the same structure that we have found in Sr_2_ZnIrO_6_ in the 673–873 K range, as described above. The LeBail refinements^21^ of a diffraction pattern of Sr_2_ZnIrO_6_ before and after the phase transition can be found in Figure 6.

**Figure 6:**
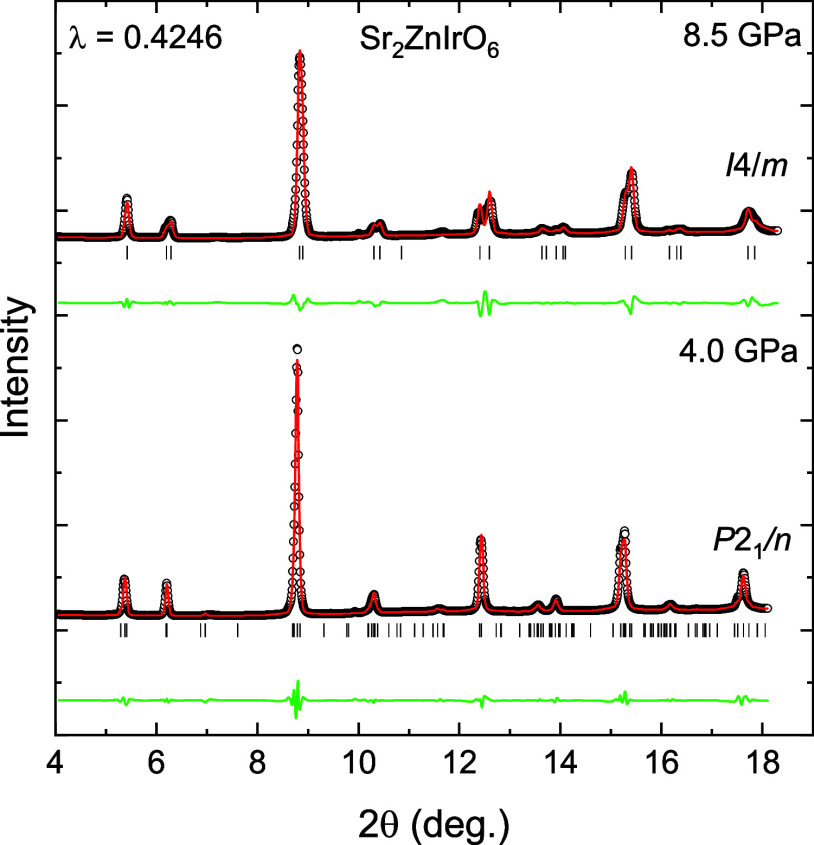
Le Bail refinement of an X-ray diffraction pattern before (4.0(1) GPa) and after (8.5(1) GPa) the phase transition at 7.5(1) GPa of Sr_2_ZnIrO_6_. The continuous red and green lines are the calculated pattern and the difference between the experimental pattern and the calculated pattern, respectively. The vertical lines mark the position of the reflections.

The quality of the Le Bail refinements confirms the symmetry of the crystal structure of the high-pressure phase of S_2_ZnIrO_6_. Furthermore, the validity of the structural symmetry proposed for the high-pressure phase of S_2_ZnIrO_6_ is supported by the crossing of the enthalpy difference lines (Figure 7) between the low-pressure structure and the proposed high-pressure structure, which occurs at 6.7 GPa. The relaxed structure is identical with that shown in Figure 1c with the atomic coordinates and lattice parameters presented in Table 3. The differences in the atomic coordinates between the calculated structure *I*4/*m* and the experimentally solved one at 673 K (Table 2) are due to the different temperature and pressure conditions. Despite this, the agreement is good.

**Figure 7:**
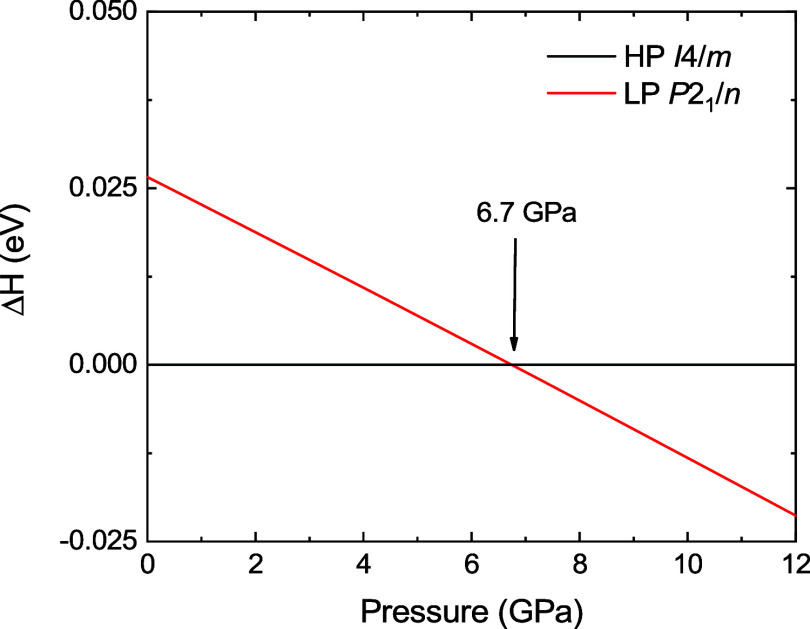
Pressure dependence of the enthalpy differences Δ*H* of the high-pressure (HP) structure found experimentally for Sr_2_ZnIrO_6_ with respect to their low-pressure (LP) phase. The crossing between the continuous black (LP) and red (HP) lines indicates the theoretical prediction of the phase transition onset also indicated by the arrow.

**Table 3:** Calculated Lattice Parameters and Atomic Coordinates for the Structure of the High-Pressure Phase of Sr_2_ZnIrO_6_ at 7.5(1) GPa

calculated high-pressure phase of Sr_2_ZnIrO_6_				
7.5 GPa space group *I*4/*m* (87)				
*a* = 5.42429 Å; *c* = 7.91897 Å; *V* = 233.000 Å^3^				
Sr	4d	0	0.5	0.25
Zn	2a	0	0	0.5
Ir	2b	0	0	0
O1	4e	0	0	0.257929
O2	8h	0.303982	0.207877	0

It is worth noting that the enthalpy differences between the low-pressure and the high-pressure phases of Sr_2_ZnIrO_6_ are only 0.025 eV at ambient pressure, which can be explained considering that the difference between the *a* and *b* lattice parameters in Sr_2_ZnIrO_6_ is only 0.2% and the tilting angle ϕ along the [001] direction is 9.16°, a value relatively small considering the remaining members of the family. As shown in Figure 7, the results confirm the structural assignment from the current XRD study, corroborating the occurrence of a *P*2_1_/*n* to *I*4/*m* phase transition in Sr_2_ZnIrO_6_ at 7.5(1) GPa.

The high-pressure-induced structural phase transition results in the expansion of the *c* lattice parameter by 1.4% (Figure 8) accompanied by a small contraction of *a* and *b* lattice parameters by 0.4%. The expansion of the *c* axis can be easily observed at Figure 5 with the abrupt change to a lower two-theta angle of the (002) reflection. The contraction of the *a* axis and the expansion of the *c* axis seem to compensate, and the unit-cell volume does not show any discontinuity in the phase transition. Hence, the whole pressure range including the low-pressure and the high-pressure phases can be fitted with the same second-order Birch–Murnaghan equation of state.^22,23^

**Figure 8:**
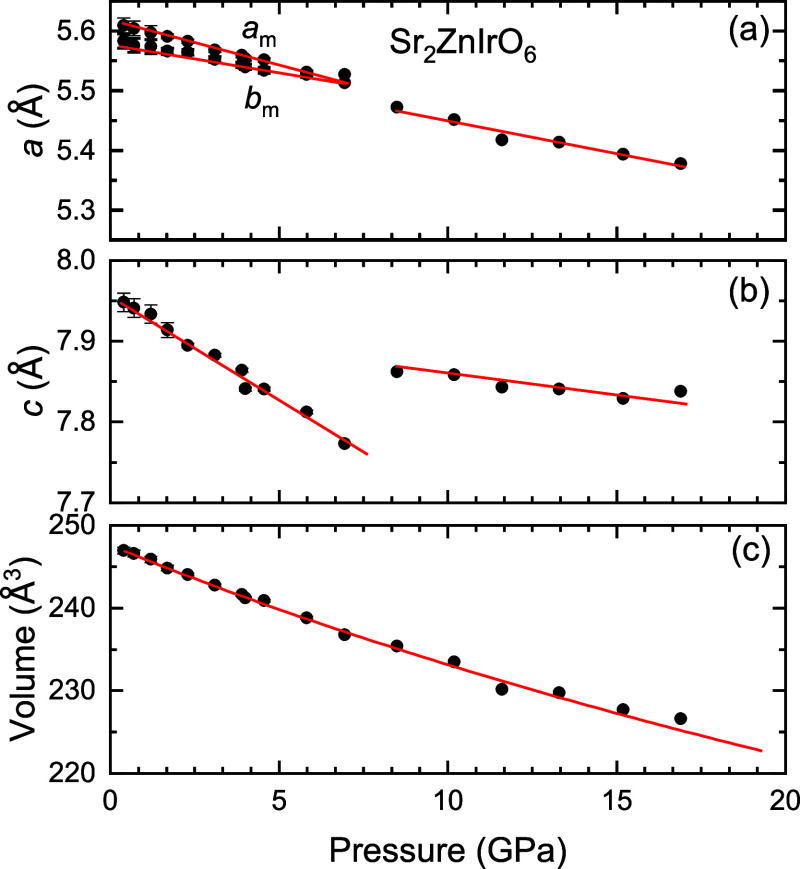
Pressure evolution of the (a) *a*, *b*, and (b) *c* lattice parameters and (c) unit-cell volume of Sr_2_ZnIrO_6_ before and after the phase transition at 7.5(1) GPa. In (a), *a*_m_ and *b*_m_ denote the lattice parameters of the low-pressure (LP) monoclinic phases that merge after the phase transition. The red lines are straight-line fits to the lattice parameters and a fit to a second-order Birch–Murnaghan equation of state for the volume. The monoclinic angle β of the LP is not included because it maintains with pressure its value 90.2(1)° within our experimental resolution.

The axial compressibility κ_*i*_ of Sr_2_ZnIrO_6_ is presented in Table 4. Considering that the monoclinic angle remains close to 90° under compression during the low-pressure phase, we can assume that the directions of compressibility coincide with the lattice axial directions in both low-pressure and high-pressure phases.

**Table 4:** Experimental Axial Compressibility $\kappa_{x}=-\frac{1}{x}\frac{\partial x}{\partial p}$ of Sr_2_ZnIrO_6_ in Both Phases

	LP	HP
κ_*a*_ (GPa^–1^)	0.0025(2)	0.0020(2)
κ_*b*_ (GPa^–1^)	0.0008(2)	
κ_*c*_ (GPa^–1^)	0.0030(5)	0.0005(1)

Since the phase transition keeps the same axes, in Table 4, we can see that the compressibility of the lattice parameters after the phase transition decreases as expected in a denser structure. The effect is dramatic in the case of κ_*c*_ with a decrease in compressibility of 1 order of magnitude from 0.0030(5) GPa^–1^ to 0.0005(1) GPa^–1^ in the high-pressure tetragonal phase. The reason is that in the phase transition, the Ir–O–Zn bonds align along the *c*-axis to get rid of the monoclinic zigzag alignment. This makes the [001] direction in the tetragonal phase rather incompressible.

Table 5 shows the bulk moduli of the low- and high-pressure phases of Sr_2_ZnIrO_6_, derived from fitting their volume–pressure curves (Figure 8) to a second-order Birch–Murnaghan equation of state.

**Table 5:** Experimental and Theoretical Bulk Moduli of the Low-Pressure (LP) and High-Pressure (HP) Phases of Sr_2_ZnIrO_6_ after Fits to Second-Order Birch–Murnaghan Equation of State as Shown in Figure 8

	experimental		calculations	
	LP phase	HP Phase	LP Phase	HP Phase
	*K*_0_ (GPa)	*K*_0_ (GPa)	*K*_0_ (GPa)	*K*_0_ (GPa)
Sr_2_ZnIrO_6_	148(7)	148(7)	154(1)	150(2)

The agreement between the experiment and the calculation is reasonable, considering the uncertainty. Experimentally, the pressure dependence of the unit-cell volume is fitted with one single second-order Birch–Murnaghan equation of state^22,23^ giving rise to an average bulk modulus of 148(7) GPa, which is well reproduced by the calculated bulk moduli of both low-pressure 154(1) GPa and high-pressure 150(2) GPa phases.

Although the evolution of the phase transition cannot be explored experimentally in detail due to the lack of reliable Rietveld refinements, the similarity of the experimental and calculated lattice parameters, and, in particular, of the Ir–O–Zn tilting angle ϕ, allows us to draw conclusions from the pressure dependence of ϕ along the phase transition. The pressure dependence of the three Ir–O–*B* tilting angles is shown in Figure 9.

**Figure 9:**
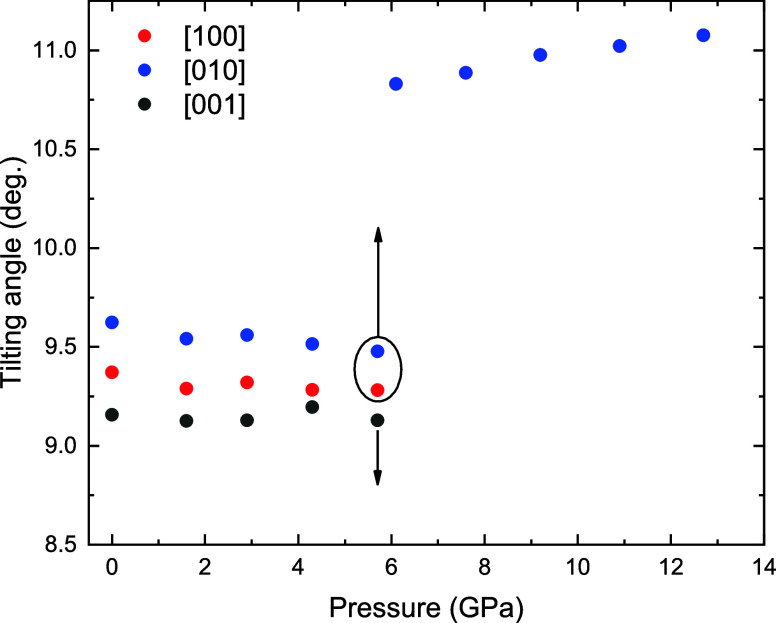
Theoretical pressure dependence of the IrO_6_ and ZnO_6_ polyhedral tilting determined by the Ir–O–Zn tilting angle ϕ along the three crystallographic axes of Sr_2_ZnIrO_6_. The alignment of the Ir–O–Zn is only perfect along the [001] direction. ϕ[001] goes to 0° at the phase transition and ϕ[100] = ϕ[010] due to the tetragonal symmetry of the high-pressure phase.

The values of the three tilting angles ϕ are very similar and exhibit a consistent linear decrease under compression causing the structure to evolve toward higher symmetry.

At 7.5(1) GPa, during the phase transition, a significant discontinuity occurs in the *c*-axis (Figure 8), which expands to accommodate a 180-degree alignment of the Ir–O–Zn bond, with no octahedral tilting, along the [100] and [010] directions, consistent with tetragonal symmetry (ϕ[100] = ϕ[010] = 0). Above 7.5(1) GPa, an increase in ϕ[100] under compression is observed, indicating that the external pressure induces a more distorted structure within the tetragonal symmetry.

An interesting point is that both increasing temperature and increasing pressure give rise to a structural phase transition from the ambient conditions monoclinic (*P*2_1_/*n*) polymorph of Sr_2_ZnIrO_6_ to the tetragonal one (*I*4/*m*). Temperature increase produces an expansion of the atomic distances and therefore an increase of the lattice parameters, as seen in Sr_2_ZnIrO_6_ (Figure 4). Otherwise, compression of the structure produces a reduction of the atomic distances and therefore a reduction of the lattice parameters, as we have shown for Sr_2_ZnIrO_6_ in Figure 8. These facts, also observed in other completely different systems as the quasi-skutterudite^24^ La_3_Rh_4_Sn_13_, resemble very much the structural phase transitions suffered by ferroelectrics in which temperature is the primary thermodynamic variable governing the phase transition, while pressure merely shifts the critical temperature of the transformation. Furthermore, since the *P*2_1_/*n*–*I*4/*m* transition does not exhibit any detectable change in unit-cell volume despite being a first-order phase transition, it is reasonable to propose that the present phase transition has a displacive character. Such hypothesis could be tested by the presence of a soft mode at the Brillouin zone center, as predicted by Samara et al.^25^ for systems with d*T*_c_/d*p* < 0. Unfortunately, due to the metallic character of Sr_2_ZnIrO_6_, Raman spectroscopy cannot be used to verify this possibility, leaving the hypothesis unconfirmed. Nonetheless, assuming that the *I*2/*m* is just a variation of the ambient conditions lattice only detected in the higher resolution high-temperature experiment, one could consider the temperature of the transformation from *P*2_1_/*n* to *I*4/*m* as *T*_c_ = 673 K. Considering that at ambient temperature the onset of the phase transition is 7.5(1) GPa, we could tentatively obtain a pressure derivative of the *T*_c_ of d*T*_c_/d*p* = (673–294)/7.5 ∼ −51 K/GPa for Sr_2_ZnIrO_6_. This is just a rough stimulation since an accurate determination of this value would require performing a high-temperature XRD study with smaller temperature steps.

## 4 Conclusions

The powder X-ray diffraction study presented here on Sr_2_ZnIrO_6_ led us to find up to three additional polymorphs for this compound. Both high pressure and high temperature induce a symmetrization of the structure mostly governed by an increase of the angle of the alternating zigzag Ir–O–Zn chains along the *c*-axis with the $\hat{IrOZn}$ angle changing from 153° to 180° either at 673 K or at 7.5(1) GPa. The structural sequence at high pressure is *P*2_1_/*n* → *I*4/*m* (at 7.5(1) GPa), whereas the sequence at high temperature is *P*2_1_/*n* → *I*2/*m* → *I*4/*m* → *Fm*3̅*m* (at 473, 673, and 873 K, respectively). Curiously, the tetragonal *I*4/*m* structure emerges both above 7.5(1) GPa and at 673 K, despite the opposing effects of pressure (compression) and temperature (expansion) on the lattice. Considering the intermediate *I*2/*m* phase as a mere distortion of the initial monoclinic *P*2_1_/*n* structure, this observation suggests a displacive transition. In such a case, *P*2_1_/*n* → *I*4/*m* would be only temperature driven with a pressure dependence of its critical temperature of d*T*_c_/d*p* = −51 K/GPa. Anyway, an accurate determination of this value as well as the determination of the phase transition onsets at high temperature would require an additional high-temperature X-ray diffraction study of Sr_2_ZnIrO_6_ with a more dense sampling with temperature. The structural sequence at high temperature perfectly coincides with that previously found in Sr_2_NiIrO_6_. However, while Sr_2_ZnIrO_6_ becomes more regular under compression transforming from the starting monoclinic *P*2_1_/*n* structure to the tetragonal *I*4/*m* one, Sr_2_NiIrO_6_ has been proposed in a previous work^5^ to transform to an unsolved triclinic structure. Given the similarities found at high temperature and the low hydrostaticity conditions at which the previous study on Sr_2_NiIrO_6_ was carried out, further high-pressure structural studies on Sr_2_NiIrO_6_ are needed.
